# Temporal clustering of Kawasaki disease cases around the world

**DOI:** 10.1038/s41598-021-01961-5

**Published:** 2021-11-19

**Authors:** Jennifer A. Burney, Laurel L. DeHaan, Chisato Shimizu, Emelia V. Bainto, Jane W. Newburger, Roberta L. DeBiasi, Samuel R. Dominguez, Michael A. Portman, Marian Melish, Andras Bratincsak, Marianna Fabi, Elena Corinaldesi, Jeong Jin Yu, Paul Gee, Naomi Kitano, Adriana H. Tremoulet, Daniel R. Cayan, Jane C. Burns, the Kawasaki Disease Climate Study Group, Emily Ansusinha, Pei-Ni Jone, Michelle Hite, Heather R. Heizer, Marsha S. Anderson, Valentina Pavan, Laura Andreozzi, Waverley Gee, Hiroyuki Suzuki

**Affiliations:** 1 School of Global Policy & Strategy, University of California San Diego, La Jolla, CA USA; 2 Scripps Institution of Oceanography, University of California San Diego, La Jolla, CA USA; 3 Department of Pediatrics, UCSD School of Medicine, University of California San Diego and Rady Children’s Hospital San Diego, 9500 Gilman Dr., La Jolla, CA 92037 USA; 4 Department of Cardiology, Boston Children’s Hospital, Boston, MA USA; 5 Department of Pediatrics, Harvard Medical School, Boston, MA USA; 6 Division of Pediatric Infectious Diseases, Children’s National Hospital, Washington, DC USA; 7 Department of Pediatrics, The George Washington University School of Medicine and Health Sciences, Washington, DC USA; 8 Department of Pediatrics, University of Colorado School of Medicine, Denver, CO USA; 9 Department of Pediatrics, Seattle Childrens Research Institute, University of Washington School of Medicine, Seattle, WA USA; 10 Department of Pediatrics, John A. Burns School of Medicine, University of Hawaii, Honolulu, HI USA; 11 Pediatric Emergency Unit, Medical and Surgical Sciences Department, S.Orsola-Malpighi Hospital, University of Bologna, 40138 Bologna, Italy; 12 Pediatric Department, Ramazzini Hospital, Carpi, 41012 Modena, Italy; 13 Pediatric Cardiology Division, Department of Pediatrics, Asan Medical Center, University of Ulsan College of Medicine, Seoul, Korea; 14 Emergency Department, Christchurch Hospital and University of Otago, Christchurch, New Zealand; 15 Research Center for Community Medicine and Department of Public Health, Wakayama Medical University School of Medicine, Wakayama, Japan; 16 Arpae-SIMC, Hydro-Meteo-Climate Service of the Regional Agency for Prevention, Environment and Energy (ARPAE), viale Silvani 6, 40122 Bologna, Italy; 17 Wellington School of Medicine, University of Otago, Wellington, New Zealand; 18 School of Medicine, Wakayama Medical University, Wakayama, Japan

**Keywords:** Cardiology, Epidemiology

## Abstract

In a single-site study (San Diego, CA, USA), we previously showed that Kawasaki Disease (KD) cases cluster temporally in bursts of approximately 7 days. These clusters occurred more often than would be expected at random even after accounting for long-term trends and seasonality. This finding raised the question of whether other locations around the world experience similar temporal clusters of KD that might offer clues to disease etiology. Here we combine data from San Diego and nine additional sites around the world with hospitals that care for large numbers of KD patients, as well as two multi-hospital catchment regions. We found that across these sites, KD cases clustered at short time scales and there were anomalously long quiet periods with no cases. Both of these phenomena occurred more often than would be expected given local trends and seasonality. Additionally, we found unusually frequent temporal overlaps of KD clusters and quiet periods between pairs of sites. These findings suggest that regional and planetary range environmental influences create periods of higher or lower exposure to KD triggers that may offer clues to the etiology of KD.

## Introduction

Kawasaki disease (KD) is the most common cause of acquired heart disease in children in the developed world, yet its etiologies remain obscure after almost half a century of research. The distribution of cases throughout the year has a distinct seasonal pattern in the northern hemisphere with a peak in case numbers in the winter/spring months and a second, lesser peak in mid-summer^1,2^. Additionally, we recently demonstrated clustering of cases that occurred at higher temporal frequency. This clustering deviated from a random Monte Carlo distribution, potentially representing an increase in the exposure to KD triggers^3^. These clusters were associated with anomalous features in temperature and atmospheric pressure that suggested an environmental influence on the distribution of cases. In the present study, we evaluated whether similar temporal clustering of cases was occurring across a range of both northern and southern hemisphere locations. We also analyzed KD cases across sites to look for similarities and differences in the timing of clusters and of quiet periods with no KD cases. We reasoned that if large scale environmental factors were involved in the transport of potential KD triggers, we might see strong temporal correlations in KD clusters or quiet periods.

## Results

**Figure 1:**
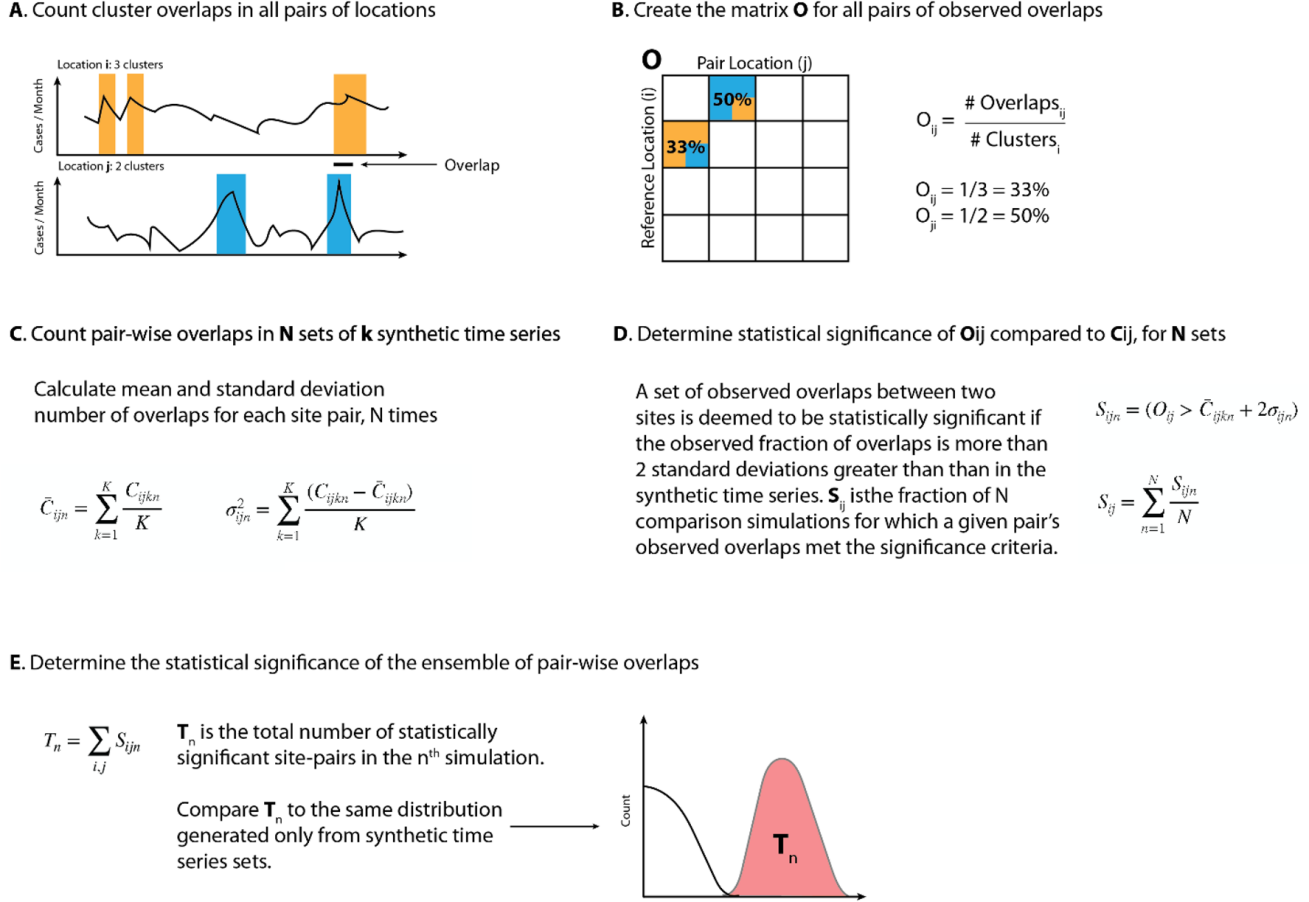
Schematic of methods used in the overlap analyses (shown here for clusters). (**A**) Clusters (periods of locally-defined high KD incidence) were defined and denoted for each site’s time series of KD onsets. Overlaps were defined between sites as contemporaneous existence of clusters in two sites, plus or minus 6 days. (**B**) We defined the matrix of pair-wise cluster overlaps, for all site-pairs. (**C**) We created 500 synthetic comparison time series for each site (shuffling cluster dates), and assessed mean and variance of cluster overlaps within pairs of sites. (**D**) We then determined statistical significance of observed site pair cluster overlaps by comparing the observed record to the mean and variance of the synthetic time series overlaps. Overlaps occurring more than 2 standard deviations from the mean of the synthetic series were considered statistically significant. We repeated (**C+D**) 500 times, and then assessed the total fraction (Sij) of times within these simulations that a site-pairs observed overlaps were deemed statistically significant. (**E**) We then tallied the total number of statistically-significant site-pairs within each simulation to assess the ensemble of overlaps. We compared this distribution to a synthetic comparison distribution in which members of our synthetic time series were compared with each other. (i.e. for step (**D**), comparing overlaps within the synthetic time series to the mean and standard deviation, as opposed to comparing the observed record). We conducted a similar process for quiet period overlaps, except that we counted the fraction of overlapping days in each quiet period. Figure 6A,B show the matrices Oij in text and Sij in color, and Fig. 6C,D show Tn.

**Figure 2:**
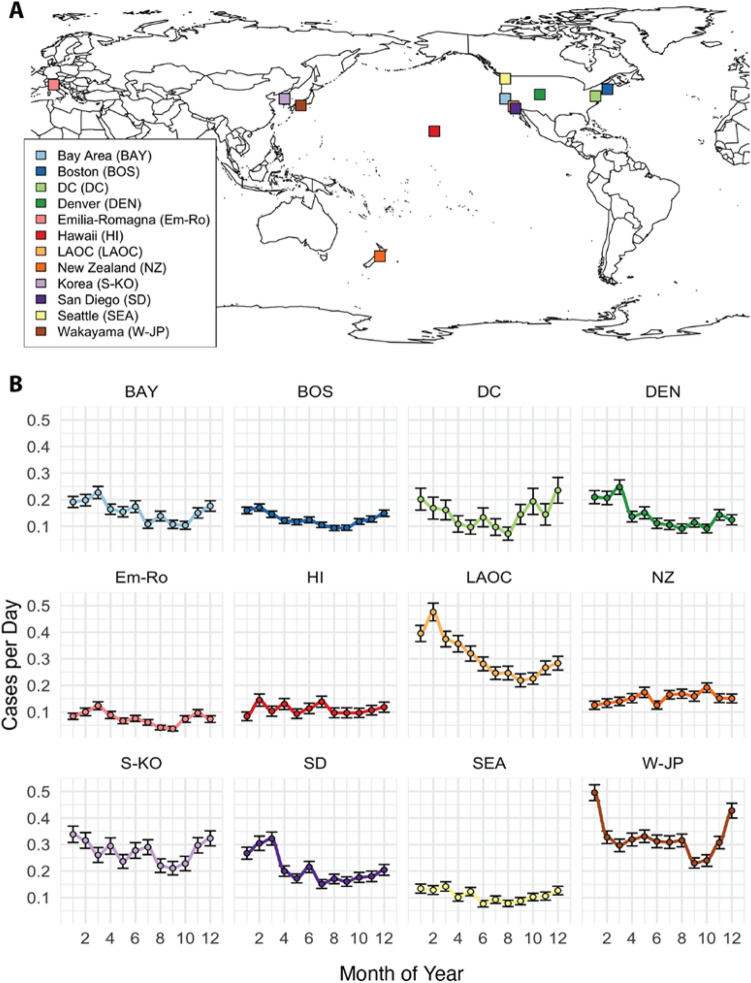
Study site locations and seasonality. (**A**) Location of 12 sites around the world. (**B**) KD incidence in each of the 12 locations displayed a distinct seasonality. Mean values ± 1 standard error are shown. Correlations of seasonal incidence across sites (i.e., the information shown in (**B**)) are shown in Supplemental Fig. S1.

**Figure 3:**
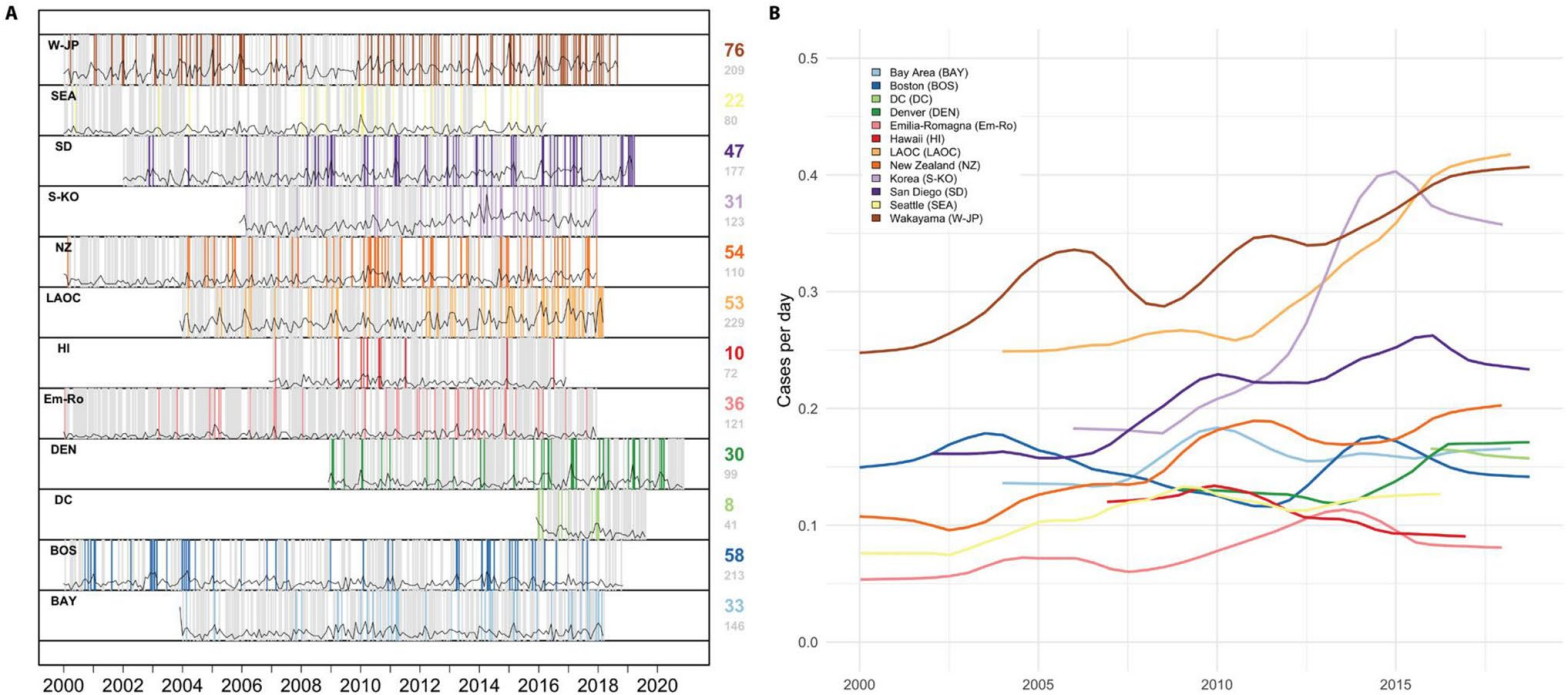
Temporal structure of clusters, quiet periods, and trends. (**A**) Shorter time periods of higher KD incidence (clusters in color) and periods of quiet (no KD onsets, grey) that occurred in patterns that differ from what would be expected by chance, accounting for seasonality (Fig. 2B) and any long-term linear trends. (**B**) A 5-year moving average shows that sites also experienced different dynamics over time with the incidence rising in most locations, except for Hawaii, Washington DC, and Boston. Correlations of rolling trends across sites (i.e., the information shown in (**B**)) are shown in Supplemental Fig. S1.

Our analysis focused on time series records of KD onsets both within and across collaborating sites around the world (see “Methods” section, Fig. 1 for summary). The KD sites in the United States included those with 35 to 100 KD patients each year and ranged from Honolulu to Boston. International sites included Japan, South Korea, Italy, and New Zealand (Table 1, Fig. 2A) Seasonality was assessed and showed a winter peak for most sites (New Zealand in austral winter, the northern hemisphere sites in boreal winter) with the exception of Hawaii (Fig. 2B). In each site, we calculated local cluster and quiet period definitions (“Methods” section, Fig. 1, Fig. 3A). Although the total numbers of clusters varied slightly depending on the choice of percentile for the definition, our chosen definitions revealed that 12–28% of KD patients occurred in these high-density clusters (Table 1). While longer-term trends and seasonal variation contributed to increased likelihood of a high-density cluster occurring, these two factors did not fully explain the time series structure. Interestingly, the trends varied across sites: in six of the locations, KD incidence has risen in recent years (Fig. 3B), while three of the sites showed flat or slightly declining levels, most notably Hawaii.

**Table 1:** Summary of sites included in the study, number of patients, and local cluster and quiet period definitions.

Location	Patients	Years	Local cluster definition (# of KD cases in 7 days)	Number of clusters	# of patients in clusters	Fraction of patients in clusters	Local quiet period definition (# days with no KD cases)	Number of quiet periods
Bay Area, CA USA	822	16	4	33	180	0.21	9	146
Boston, MA USA*	1017	18	**4**	43	223	0.22	**15**	96
DC, USA	193	5	**4**	8	44	0.23	9	41
Denver CO, USA	629	13	4	30	179	0.28	11	99
Emilia-Romagna, IT	502	18	3	36	139	0.28	16	121
Hawaii, USA	403	11	**4**	10	48	0.12	14	72
Los Angeles, CA USA	1603	16	6	53	458	0.29	5	229
New Zealand	1008	19	4	54	274	0.27	**14**	110
Seoul, Korea	1205	13	6	31	257	0.21	**7**	123
San Diego, CA USA	1332	18	5	47	315	0.24	**8**	177
Seattle, WA USA	646	17	**4**	22	112	0.17	18	80
Wakayama Prefecture, Japan	2255	20	6	76	619	0.27	6	209

Bold indicates use of the 99.5th percentile as a definition for either cluster or quiet period; black indicates use of 97.5th percentile. We used the more conservative cluster definition in lower incidence areas because an integer value (e.g., 2 cases in 7 days) might span a large percentile range of the distribution of densities in a low-incidence area. We used the higher threshold to define quiet periods in higher incidence regions by a similar logic, to ensure we had used tail values when constrained to use integer values (*The full record from Boston included 1596 patients over 35 years, with 293 patients in 58 clusters, or 18%, and 213 quiet periods. For the analysis here we restricted analysis to after 2000).

**Figure 4:**
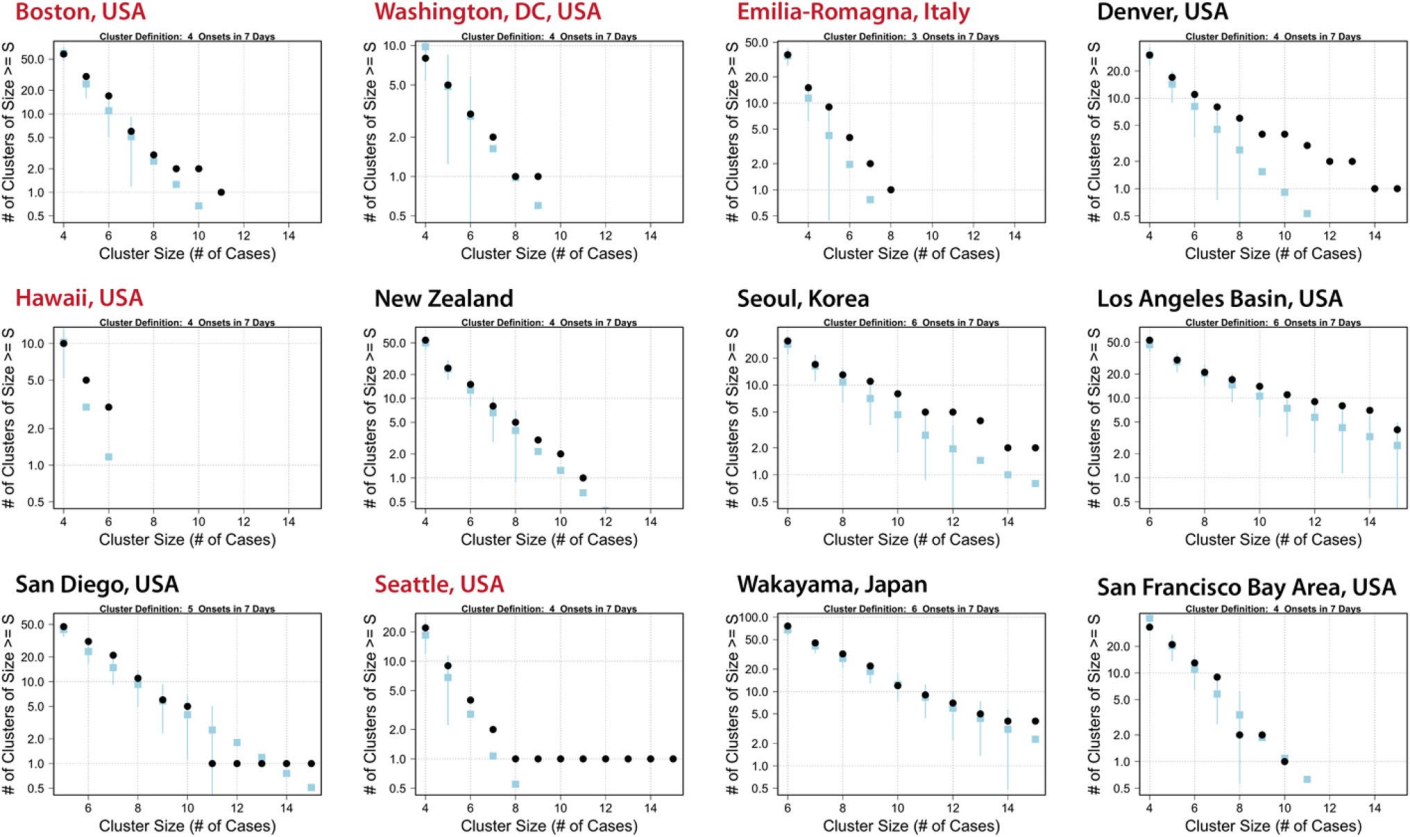
Distributions of clusters in observed KD time series (black dots) and 100 Monte Carlo time series (blue squares) with local trends and seasonality. For reshuffled time series, mean and ± 2 standard error are shown. Locations in red used the 99.5th percentile of maximum case density as the initial cluster definition; locations in black used the 97.5th percentile (Non-logarithmic version shown in Supplemental Fig. S2).

**Figure 5:**
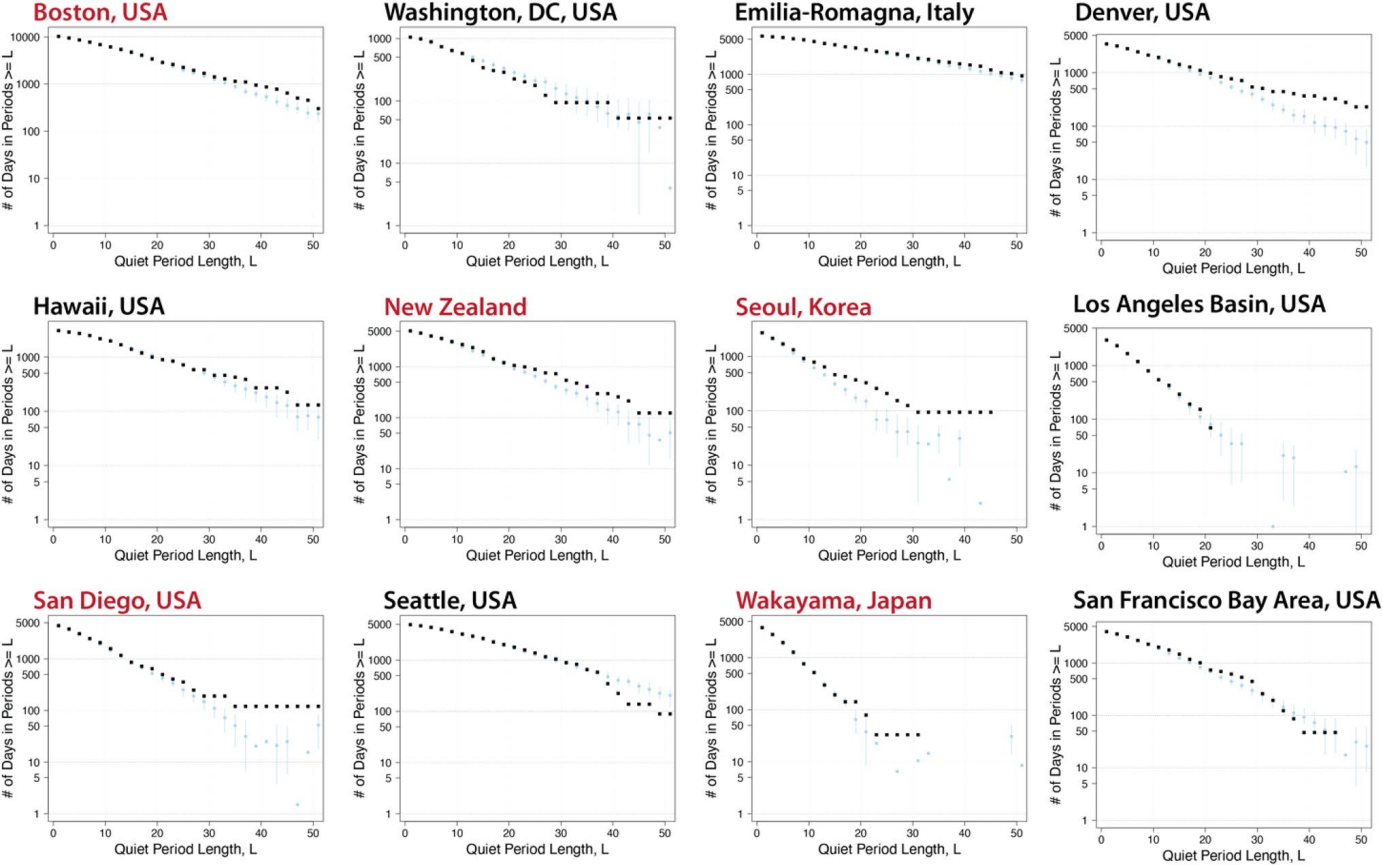
Distributions of quiet periods in observed KD time series (black dots) and 100 Monte Carlo time series (blue squares) with local trends and seasonality. For reshuffled time series, mean and ± 2 standard error are shown. Locations in red used the 99.5th percentile of maximum case density as the initial cluster definition; locations in black used the 97.5th percentile.

We compared the observed data in each location to 100 synthetic comparison time series created by randomly reshuffling onset dates while respecting local time trends and seasonality of KD incidence. For the observed data and each of these synthetic time series, we counted both KD clusters and quiet periods based on the thresholds determined in the observed data. We found that the distributions of cases and quiet periods differed between the observed KD data and the synthetic (i.e., simulated; see “Methods” section) time series at most sites (Fig. 4, Supplemental Fig. S2 for clusters, Fig. 5 for quiet periods). The divergences of observed data from comparison distributions was largest and most clear for locations with higher KD incidence—the Seoul area in Korea, Wakayama prefecture in Japan, San Diego, and New Zealand—but existed for most sites. In general, across sites, more clusters occurred in the observed data than in the synthetic time series. There were some differences across sites in how the observed cases diverged from the synthetic comparison time series: for example, in San Diego, there were more clusters of six or seven patients than would be expected at random, but in Wakayama (Japan), that divergence happened further out in the tail of the distribution (clusters of 13 or more cases).

We found similar patterns when examining quiet periods (Fig. 5). The distribution of observed quiet periods in most of the sites diverged from the distribution of quiet periods in the synthetic comparison time series. In all cases but one, the observed data had more days in longer no-KD periods than the comparison series. The exception, Washington DC, may be due to the relatively short duration of the time series, which is a less reliable sample and makes the generation of comparison series less stable. The other two sites that showed less divergence were the Los Angeles/Orange County basin, and the San Francisco Bay Area. For both of these, data were drawn from the Pediatric Health Information System (PHIS) Database, and date of onset was estimated by subtracting five days from the date of hospitalization. This may have introduced uncertainty in the quiet period definition for these locations.

**Figure 6:**
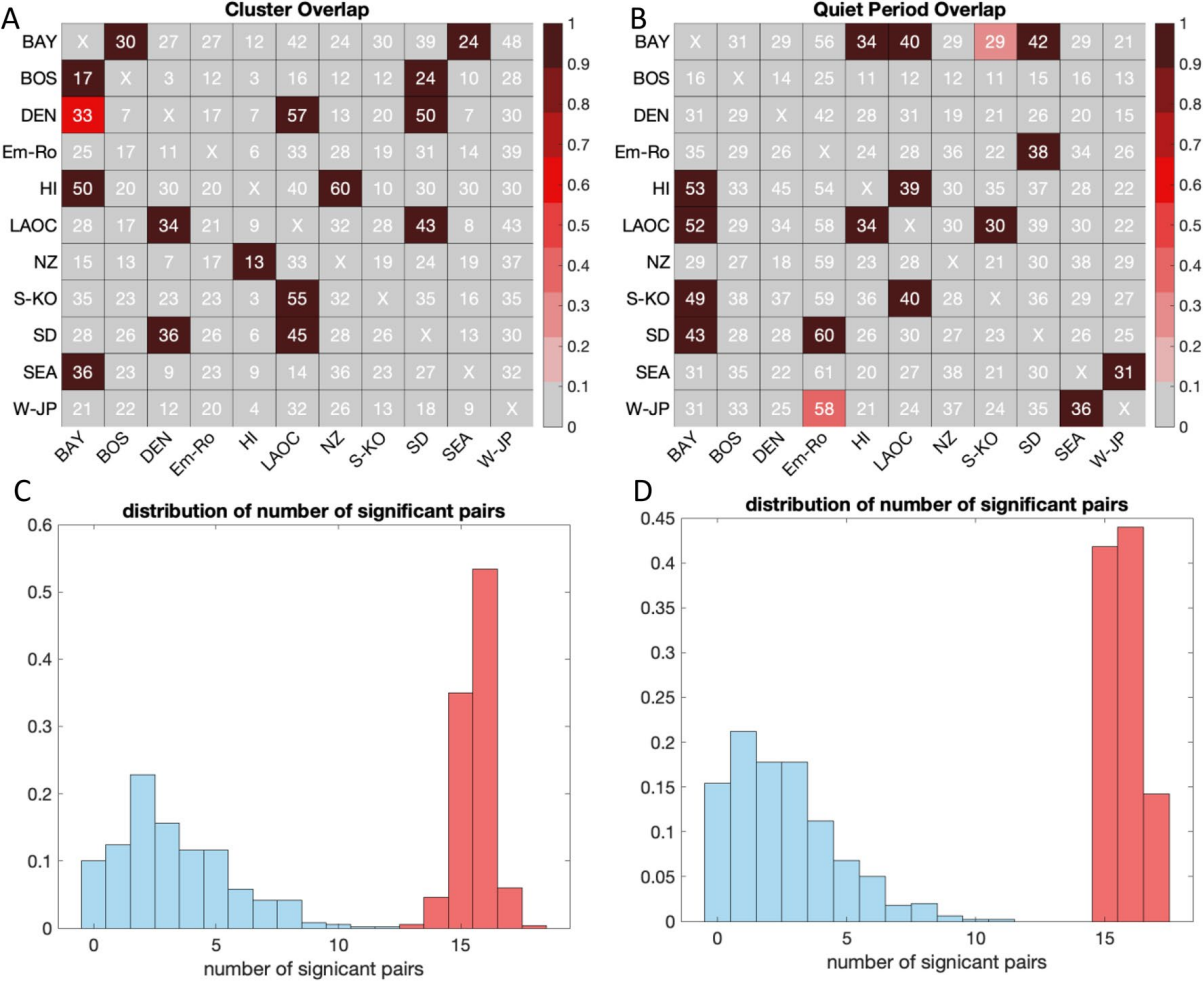
Statistics of cluster and quiet period overlaps across pairs of sites. (**A,B**) Number in each cell is the observed overlap (Oij) between KD clusters (**A**) and quiet periods (**B**) across all pairs of sites. Cell color shows the fraction of times (over 500 simulations) that a pair of sites had observed overlaps that were statistically different (> 2SD) from the simulated time series overlaps (Sij). (**C,D**) Blue distribution shows the total number of pairs of sites with statistically significant overlaps when each member of the synthetic time series is compared to the other 499; red distribution shows the total number of statistically significant site pairs when the observed record was compared to the 500 synthetic time series (i.e., the number of pairs of sites with overlaps > 2 standard deviations above the comparison, or Tn).

We calculated temporal overlaps between clusters (numbers in cells in Fig. 6A) and quiet periods (numbers in cells in Fig. 6B) at different sites. Overlaps were calculated for all pairs in reference to each site. For example, 36% of San Diego clusters overlapped with clusters in Denver but 50% of Denver clusters overlapped with San Diego clusters. Because the length of record and number of clusters differed across sites, the fraction required for a significant overlap varied from as little as 0.1 to over 0.9. The fraction of times a pair had a significant overlap is shown by the cell color in Fig. 6A,B and the distribution of the number of significant overlaps from each Monte Carlo exercise is shown in Fig. 6C,D.

Overlaps in individual pairs of sites and the total ensemble of sites in some cases suggested regional-scale coordination. (Fig. 6A). For example, San Diego and Los Angeles shared many cluster periods as did the San Francisco Bay Area and Seattle. However, other cases pointed to longer-distance connections—Hawaii and New Zealand shared significant overlap, as did the Bay Area and Boston. Interestingly, clustering at Emilia-Romagna and Wakayama Prefecture, Japan, did not exhibit significant overlaps with any of the other sites. Several of the pairs of sites had one-way overlaps (that is, the relationship was only statistically significant when referenced to one of the two sites): Hawaii-Bay Area and Seoul South Korea—Los Angeles.

The quiet periods exhibited a distinctly different set of pair-wise overlaps (Fig. 5B). San Diego exhibited quiet period overlaps with both Emilia-Romagna and the San Francisco Bay Area. Los Angeles and Hawaii had significant quiet period overlaps, as did Seattle and Wakayama Prefecture, Japan. Quiet periods in Boston, Denver, and New Zealand did not overlap significantly with any of the other sites.

For the ensemble of site pairs, we found that the total number of pair-wise significant overlaps for both clusters and quiet periods were much greater than would be expected at random (Fig. 6C,D). From the Monte Carlo tests, 14 to 17 of the pairs of observed records exhibited statistically significant number of cluster overlaps compared to approximately 2 to 6 pairs of the synthetic records (Fig. 6C), and 15 to 17 of the pairs of observed records exhibited statistically significant number of quiet period overlaps compared to approximately zero to seven pairs of the synthetic records (Fig. 6D).

## Discussion

The analysis of data from worldwide locations demonstrates that clustering of KD cases and quiet periods with no KD cases are generalized phenomena occurring in both the northern and southern hemispheres. These periods of high and low KD density occurred more than would be expected from trends in KD incidence and seasonal fluctuations alone. Furthermore, clusters and quiet periods occurred synchronously between regional and more distant locations more often than expected by chance. We view cluster and quiet periods within single sites, and overlaps between sites, as important because they suggest the presence or absence of the KD agent or agents. However, the observation that the pairings of cluster and quiet period overlaps differed across sites suggests that a combination of processes drive exposure to the agent(s) that trigger KD. These observations should be used to focus research on the potential exposures that are triggering these clusters in genetically susceptible children.

To create this novel dataset, over a decade of patient records from an international array of hospital units were reviewed to capture dates of fever onset, as opposed to date of hospitalization or date of diagnosis. With these data we created a unique cluster and quiet period definition for each site. This permitted a large-scale comparison of KD onsets that uncovered coherent patterns of clusters and quiet periods across sites. This is a novel example of people-as-sensors for environmentally-associated phenomena that act as KD triggers. Additionally, these findings indicate that collecting and analyzing data on a global scale are vital to develop a framework for understanding KD etiology.

This work builds on previous studies by other investigators. Temporal and spatial clustering of KD cases was first described by Nakamura and colleagues using the nationwide surveys that are conducted every 2 years in Japan^4^. They reported that spatial clustering occurred in both epidemic and non-epidemic years and was more pronounced in urban areas. A more detailed study using the Kulldorff spatial scan statistic identified high incidence years in Tokyo and Kumamoto regions of Japan based on 73,758 KD patients^5^. The first Western report of temporospatial clustering described spatial clustering of KD cases at distances of 3 km and time scales of 3–5 days in San Diego County^6^. More detailed analysis of this same region linked KD clusters to large-scale climatic conditions^3^. Spatiotemporal clustering of KD cases has been reported by other groups using different time scales and data from administrative databases for KD hospitalizations^7^. Temporal KD clusters were identified across Canada at time scales spanning several months. In a further analysis by this same group, bursts of KD activity were reported in discrete locations across Canada^8^. A single center study of 263 KD patients from upstate New York used the same Kulldorff spatial scan statistic and found no significant clustering^9^. Burns et al. previously demonstrated that clinical sub-phenotypes of KD also clustered temporally. Collection of detailed clinical and laboratory data could make the analysis of clusters even more informative.

A strength of the current study was the use of actual dates of fever onset to detect temporal clusters on a weekly time scale for most sites. This can facilitate comparison of KD clusters to environmental and climatic features to probe the etiologic triggers of KD. In this analysis, we used a 6-day window to evaluate the co-occurrence of clusters across sites, which was designed to allow for possible long-range atmospheric transport of a KD agent. The brief occurrence of clusters with a time scale that is typically ten days or less seems more consistent with an airborne agent that is driven by fluctuating atmospheric circulation patterns than a transmissible agent. The existence of few day to seasonal anomalous, hemispheric-scale atmospheric circulation patterns provides a possible transport mechanism for potential KD agents. Coordination of clusters across smaller spatial scales (e.g., San Diego-Los Angeles-Denver or Bay Area-Seattle) may point to regional environmental drivers, whereas coordination at longer distances (e.g., Hawaii-New Zealand) may point to planetary scale teleconnections or long-range transport-driven exposures. Work by Rodo and colleagues revealed an atmospheric circulation bridge across the mid-latitude Pacific Ocean that provided a potential mechanism for both the major epidemics in Japan and linked interannual fluctuations in KD cases in Japan and San Diego^10,11^. Thus, long-range transport of particles that act as KD triggers may explain the clusters and quiet periods described in this study.

Limitations to the current study include the short duration of time series from some sites, small numbers of cases, and a sparse set of global records. In the absence of a gold standard diagnostic test for KD, we cannot exclude that some patients with other diagnoses were included in our time series. This is a regrettable challenge for all research on KD. To minimize this possibility, data for this study was provided by health care professionals who are experienced and skilled in the diagnosis of KD. Use of the PHIS data for two of the regions has all the limitations associated with use of an administrative database including coding errors and missed cases. Our analysis also focused on unusually high levels of KD (clusters), or unusually long absences of KD (quiet periods), and did not address less concentrated KD occurrences, which are in fact the majority of cases in every site. As such, it may be interesting in the future to analyze the “intermediate” state between cluster and quiet, and its associations and coincidences across time and space. This type of study would best be conducted with higher density of sites coordinating their data collection and reporting. Increased surveillance including observations of various clinical measures would also enable a more in-depth probing of spatial scales for KD cluster and quiet period coincidence including KD sub-type occurrence.

In conclusion, the widespread occurrence of temporal clustering and quiet periods of KD and its unusual degree of synchrony across regions exhibited in this international dataset goes beyond the well-established seasonality of the disease. This suggests large scale environmental features are associated with exposure to the triggers for KD.

## Methods

### Subjects

The date of onset of fever for each case was collected from existing databases of prospectively collected data (US sites: San Diego, Boston, Seattle; International site: Wakayama Prefecture, Japan), from retrospective medical record review (US sites: Denver, Washington, DC, Hawaii; International sites: Seoul, South Korea, Emilia-Romagna, Italy), or from national government health record databases (New Zealand). All patients met the American Heart Association (AHA) 2017 case definition for either complete or incomplete KD^12^. The study was reviewed and approved by the Institutional Review Board of the University of California San Diego (UCSD # 170045) who provided a waiver of consent as no protected health information was collected or disclosed for the performance of this study. All methods were carried out in accordance with relevant guidelines and regulations. In addition, we included cases in the Pediatric Health Information System (PHIS) database for hospitals in the Los Angeles Basin (Los Angeles, Orange County, Long Beach) and in the San Francisco Bay Area (Palo Alto, Oakland). For the Los Angeles Basin and San Francisco Bay cases, we did not have date of onset, but instead estimated a date of onset by subtracting 5 days from date of diagnosis. These sites were included to test for potential temporal coordination between geographically proximate sites with large numbers of KD patients on the West Coast, with the understanding that results should be interpreted with awareness that the date of onset was estimated.

### Cluster and quiet period definitions

We applied the clustering methodology as previously published to the data from participating sites^13^. Briefly, we defined the first day of fever as the onset of the illness. We then took the entire time series of KD onsets for each individual site, and for each date in the time series, counted the maximum number of cases over all 7-day windows containing that date. We selected the 97.5th or the 99.5th percentile value (the lower threshold for higher-density KD sites, and the higher threshold for lower-density sites) as the starting definition for a temporal cluster at that location. We then grouped all consecutive days that met the threshold condition (e.g., for the San Diego record, 5 KD patients with symptom onset in 7 days) as an individual cluster. Although the clusters that we defined started from a seed definition based on the total distribution of cases in the time series, they could extend to any number of days. That is, although the starting definition of a cluster might be 5 or more KD patients with onsets in a 7-day window, the final cluster contained the full window of consecutive days that met that threshold density (e.g., 11 cases distributed over a period of 15 days).

To test whether observed clustering differed from what would be expected based on longer-run time trends in incidence and well-established seasonality, we generated 100 synthetic control time series of equal-N onset dates (Monte-Carlo simulation) that adhered to the local seasonality and long-term trends. Specifically, for each site, we generated a set of probability weights for the date range of the observed data that were comprised of any observed trend and the average monthly pattern of KD occurrence (seasonality) in that location. We then selected N dates (with replacement) from the date range, using those weights, to generate a comparison time series with the same number of cases as in the observed data. We repeated this 100 times and tallied the clusters in these synthetic records using the same method as in the observed KD time series. We then calculated distributions of clusters in these control time series and compared them to the observed data.

We additionally defined quiet periods based on the local case density distributions. For all days in each time series that had no KD onsets, we calculated the maximum time window with no cases containing that day. We took the distribution of the length of these “quiet windows” and selected the 97.5th or the 99.5th percentile value as our threshold, and then denoted all consecutive no-KD days in windows at or above this threshold as quiet periods. To test whether these occurred in manner different than would be expected at random, we used the same synthetic control time series described above and tallied the distributions of quiet periods in each. We then compared the quiet period distributions of these control time series to the observed data.

### Overlap statistics

We calculated overlaps in cluster and quiet periods across sites by both raw numbers and percentages. To account for improved awareness and diagnosis of KD over time, we restricted our study across sites to post-2000, when increased incidence of KD in these sites was less likely due to improving clinician diagnostic skill.

Figure 1 schematically illustrates our methods for assessing overlaps. For each pair of sites, the cluster overlap was defined as the fraction of the number of clusters at one site that occurred within ± 6 days of a cluster at the other site. Under this definition, the total number of overlaps obtained from any two sites depended on which site was the reference (see Fig. 1 for methods and Fig. 5 for specific pairs; for example, a larger fraction of Boston clusters (BOS) overlapped with Bay Area clusters (BAY) than the reciprocal comparison). Quiet period overlaps were defined slightly differently: an overlap of at least 1 day between sites was required, and the overlap was weighted by the total length of the temporal overlap. The 6-day window for cluster overlap was included for the following reasons: (a) to account for the uncertainty in onset date from two of the sites, (b) to account for the variability in the individual site definition of a cluster, and (c) to allow for the time delay in exposure that might occur under long-range atomospheric transport of a KD agent between sites. Such a window was not included for quiet period overlaps because their endpoints were more clearly defined by the occurrence of any KD case. To test if these overlaps were different than what would be expected by chance we compared the observed coincidence of clusters and quiet periods across sites to overlaps from a set of 500 synthetic control time series that respected the seasonality for each site. We defined pairs of sites with statistically significant overlaps as those with an observed fraction of overlaps > 2 standard deviations above the fraction of overlaps in the synthetic control time series.

To then assess the statistical significance of the ensemble of observed overlaps (i.e., to test whether the number of pairs of sites with a significant overlap was significantly different than what would be expected at random), we performed a Monte Carlo exercise, repeating the calculation of significant overlapping pairs 500 times. While the actual fraction of overlap remained the same, the mean and standard deviation from the synthetic time series varied with each Monte Carlo. We compared the total number of significant site pairs in the observed record compared to the synthetic comparison time series, and then also compared overlaps in the synthetic comparison time series to each other (as a metric of how many spurious overlapping pairs might occur at random). We omitted Washington, DC, from this analysis because of its substantially shorter time series.

## Supplementary Information

Supplementary Figures.

## References

[1] Burns JC (2005). Seasonality and temporal clustering of Kawasaki syndrome. Epidemiology.

[2] Burns JC (2013). Seasonality of Kawasaki disease: A global perspective. PLoS ONE.

[3] Rypdal M (2018). Clustering and climate associations of Kawasaki Disease in San Diego County suggest environmental triggers. Sci. Rep..

[4] Nakamura Y, Yanagawa I, Kawasaki T (1987). Temporal and geographical clustering of Kawasaki disease in Japan. Prog. Clin. Biol. Res..

[5] Sano T (2016). Temporal and geographical clustering of Kawasaki disease in Japan: 2007–2012. Pediatr. Int..

[6] Kao AS, Getis A, Brodine S, Burns JC (2008). Spatial and temporal clustering of Kawasaki syndrome cases. Pediatr. Infect. Dis. J..

[7] Hearn J (2018). Spatiotemporal clustering of cases of Kawasaki disease and associated coronary artery aneurysms in Canada. Sci. Rep..

[8] Manlhiot C (2018). Environmental epidemiology of Kawasaki disease: Linking disease etiology, pathogenesis and global distribution. PLoS ONE.

[9] Chang A, Delmerico AM, Hicar MD (2019). Spatiotemporal analysis and epidemiology of Kawasaki disease in Western New York: A 16-year review of cases presenting to a single tertiary care center. Pediatr. Infect. Dis. J..

[10] Rodo X (2014). Tropospheric winds from northeastern China carry the etiologic agent of Kawasaki disease from its source to Japan. Proc. Natl. Acad. Sci. U.S.A..

[11] Ballester J (2019). On the interpretation of the atmospheric mechanism transporting the environmental trigger of Kawasaki Disease. PLoS ONE.

[12] McCrindle BW (2017). Diagnosis, treatment, and long-term management of Kawasaki disease: A scientific statement for health professionals from the American Heart Association. Circulation.

[13] Burns JC (2021). Temporal clusters of Kawasaki disease cases share distinct phenotypes that suggest response to diverse triggers. J. Pediatr..

