# Supplementary material for: Temporal clustering of Kawasaki disease cases around the world
Source: Sci Rep. 2021 Nov 19;11:22584. doi: 10.1038/s41598-021-01961-5 (PMC8605018; doi:10.1038/s41598-021-01961-5)

**Supplemental information**

Temporal clustering of Kawasaki disease cases around the world

Jennifer A. Burney^1^, Laurel L. DeHaan^2^, Chisato Shimizu^3^, Emelia V. Bainto^3^, Jane W. Newburger^4^, Roberta L. DeBiasi^5^, Samuel R. Dominguez^6^, Michael A. Portman^7^, Marian Melish^8^, Andras Bratincsak ^8^, Marianna Fabi^9^, Elena Corinaldesi^10^, Jeong Jin Yu^11^, Paul Gee^12^, Naomi Kitano^13^, Adriana H. Tremoulet^3^, Daniel R. Cayan^2^, Jane C. Burns^3^ and the KD Climate Study Group

^1^ School of Global Policy & Strategy, University of California San Diego, La Jolla, CA, USA

^2^ Scripps Institution of Oceanography, University of California San Diego, La Jolla, CA, USA

^3^ Department of Pediatrics, University of California San Diego and Rady Children’s Hospital San Diego, La Jolla, CA, USA

^4^ Department of Cardiology, Boston Children’s Hospital, Department of Pediatrics, Harvard Medical School, Boston, MA, USA

^5^ Division of Pediatric Infectious Diseases, Children’s National Hospital, and Department of Pediatrics, The George Washington University School of Medicine and Health Sciences, Washington DC, USA

^6^ Department of Pediatrics, University of Colorado School of Medicine, Denver, CO, USA

^7^ Department of Pediatrics, University of Washington School of Medicine, Seattle Childrens Research Institute, Seattle, WA, USA

^8^ Department of Pediatrics, John A. Burns School of Medicine, University of Hawaii, Honolulu, HI, USA

^9^ Pediatric Emergency Unit, Medical and Surgical Sciences Department, S.Orsola-Malpighi Hospital, University of Bologna, 40138 Bologna, Italy

^10^ Pediatric Department, Ramazzini Hospital, Carpi, 41012 Modena, Italy

^11^ Pediatric Cardiology Division, Department of Pediatrics, University of Ulsan College of Medicine, Asan Medical Center, Seoul, Korea

^12^ Emergency Department, Christchurch Hospital and University of Otago, Christchurch, NZ

^13^ Research Center for Community Medicine and Department of Public Health, Wakayama Medical University School of Medicine, Wakayama, Japan

**Supplemental Figure 1**: Pairwise correlations between 5-year rolling average trends across sites (i.e., the data shown in **Figure 3B**), and seasonal patterns across sites (i.e., the data shown in **Figure 2B**). Correlations are calculated on a daily basis from the components of a time series decomposition for each site.


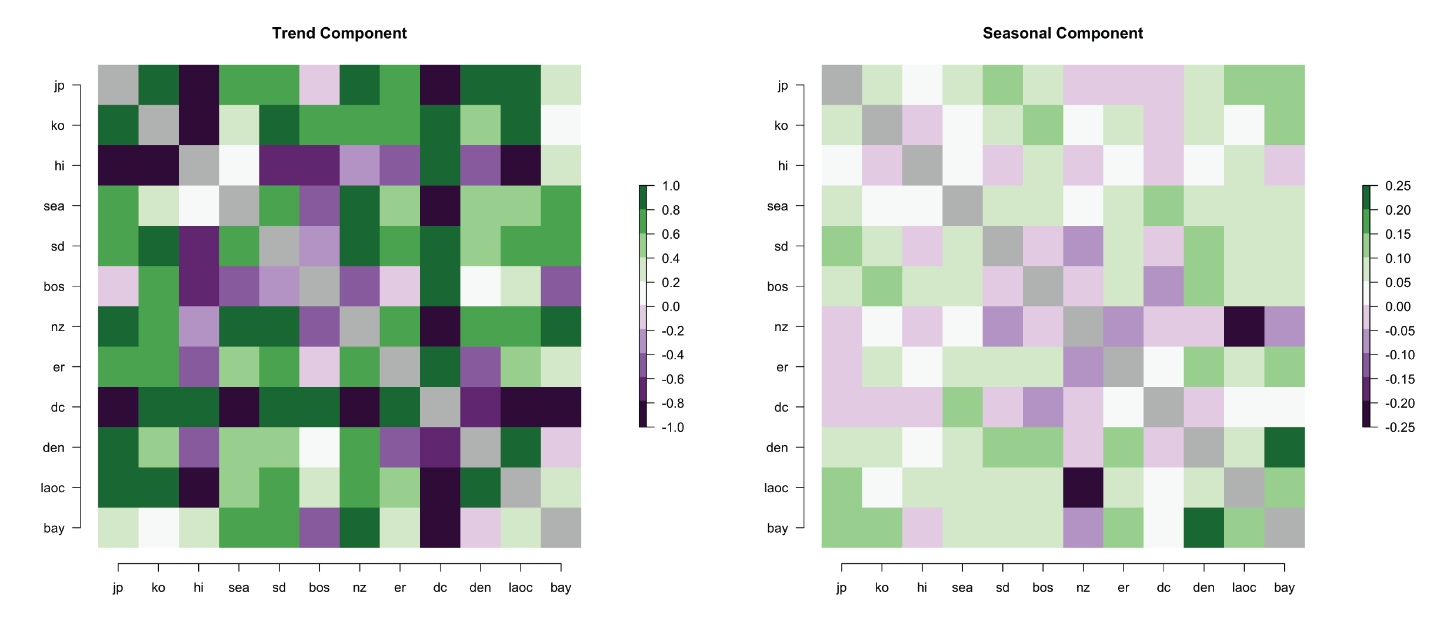


**Supplemental Figure 2**: As in **Figure 4**, but with non-logarithmic vertical axis scale.


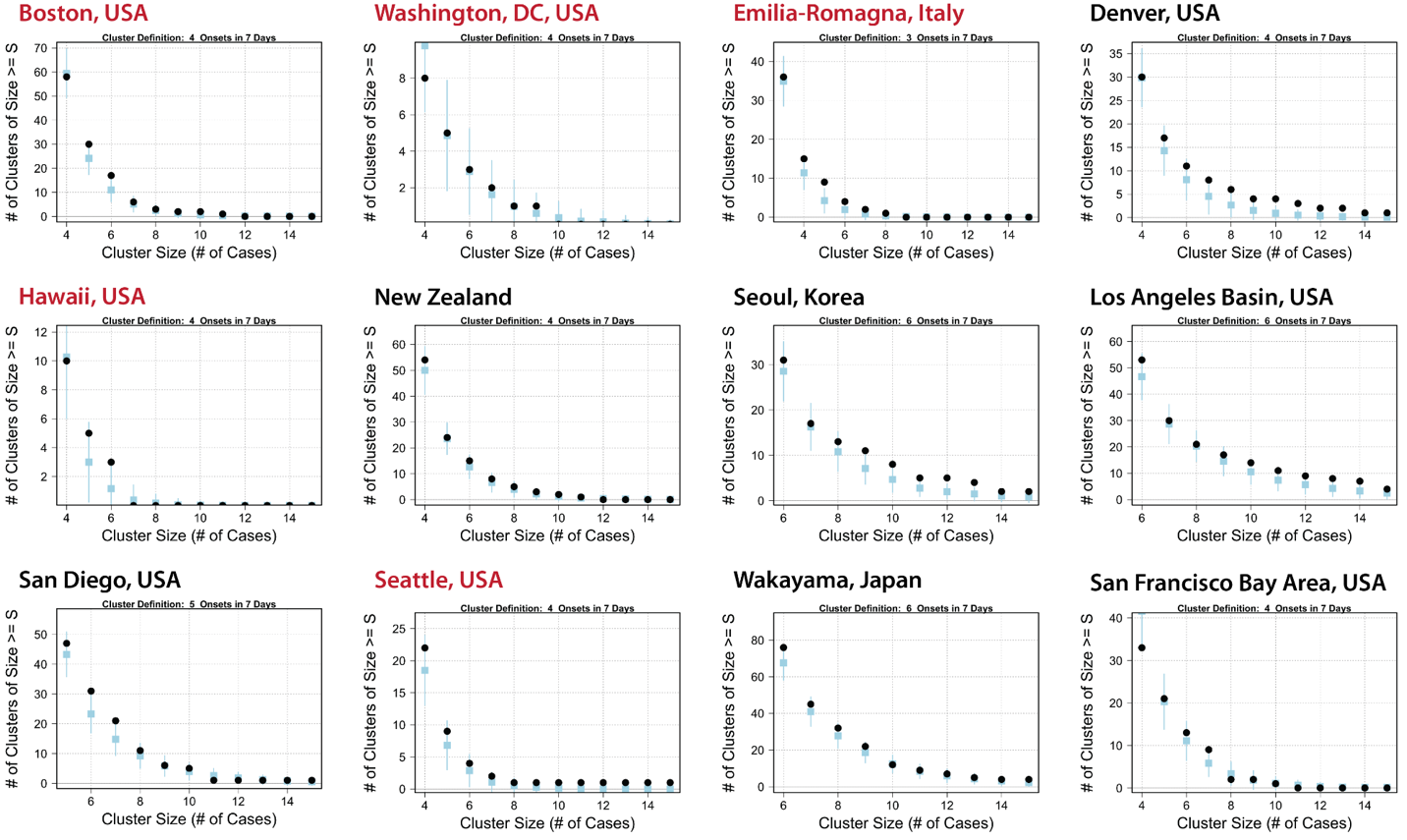

Supplement: Supplementary file 1 — Supplementary Figures. [file 41598_2021_1961_MOESM1_ESM.docx]
